# Navigating the road ahead: using concept mapping to assess Clinical and Translational Science Award (CTSA) program goals

**DOI:** 10.3389/fpubh.2025.1562191

**Published:** 2025-03-31

**Authors:** Cathleen Kane, William Trochim, Haim Bar, Andie Vaught, Heather Baker, Munziba Khan, Robin Wagner, Kristi Holmes, Keith Herzog, Jamie Mihoko Doyle

**Affiliations:** ^1^New York University Langone Health, Clinical and Translational Science Institute (CTSI), New York, NY, United States; ^2^The Brooks School of Public Policy, Cornell University, Ithaca, NY, United States; ^3^Department of Statistics, University of Connecticut, Storrs, CT, United States; ^4^Division of Clinical Innovation, National Center for Advancing Translational Sciences, National Institutes of Health, Bethesda, MD, United States; ^5^Northwestern University Feinberg School of Medicine, Northwestern University Clinical and Translational Sciences (NUCATS) Institute, Chicago, IL, United States

**Keywords:** Clinical and Translational Science Awards (CTSA), concept mapping, evaluation study, stakeholder participation, mixed-methods research, Translational Science Benefits Model (TSBM), longitudinal impact, National Institutes of Health (NIH)

## Abstract

Evaluating large-scale programs designed to transform public health demands innovative approaches for navigating their complexity and scope. The Clinical and Translational Science Awards (CTSA) Program, supported by the NIH's National Center for Advancing Translational Sciences (NCATS), represents a significant national investment with over 60 sites or “hubs” spread across the country. Assessing an initiative of this size and complexity requires measures that balance local flexibility with national coherence. To that end, this study used concept mapping, a mixed-methods approach integrating qualitative brainstorming and sorting with quantitative multidimensional scaling and cluster analysis. Participation across the CTSA was unprecedented. Over 100 evaluation stakeholders were engaged across the network of hubs, leading to the identification of more than 80 measures, which were then organized into thematic clusters that reflect a logical progression from CTSA activities to outcomes and impacts, as well as critical foundational factors such as collaboration and education. The results also revealed a pattern where long-term impacts were ranked among the highest in importance but among the lowest in feasibility, particularly for measures tied to the Translational Science Benefits Model (TSBM), a new evaluation framework gaining popularity across the CTSA. The findings of this study underscore the efficacy of concept mapping in incorporating wide-ranging perspectives, identifying areas of consensus, and informing leadership in the development of unified, data-driven evaluation frameworks —such as TSBM and/or a CTSA logic model— critical to maximizing the CTSA's transformative potential for public health.

## 1 Introduction

It has been over 17 years since the National Institutes of Health (NIH) launched the Clinical and Translational Science Awards (CTSA) Program, an ambitious set of bold initiatives (1) and national investments aimed at improving the process of transforming laboratory, clinical, and community-based discoveries into effective public health interventions (2). The CTSA program is a nationwide network of medical research institutions, referred to as “hubs”, designed to synergize infrastructure and interdisciplinary, scientific expertise to advance clinical and translational science (CTS) research. CTSA hubs facilitate translational research through targeted pilot awards, research support services, community engagement, and multidisciplinary training. In Fiscal Year 2024, the National Center for Advancing Translational Sciences (NCATS) invested more than $629 million (3) to support more than 60 hubs.

Large biomedical research investments, such as the CTSA program, require rigorous process and outcome evaluations to determine whether the program is meeting its goals and if systematic modifications are needed over time. The Foundations for Evidence-based Policymaking Act of 2018 (also known as the Evidence Act) further underscores the need for federal agencies to build evidence in support of programs and decision-making, including the CTSA program and NCATS (4). However, the CTSA program's expansive goals, diverse institutional activities, and decentralized structure create a complex evaluation environment requiring an approach that balances local flexibility with consortium-wide coherence (5). Assessments of programs with this complexity present both practical and theoretical challenges. One practical challenge, for instance, centers on the number of CTSA institutions supported (>60) that are geographically disparate. Another challenge is building consensus among multiple evaluation stakeholders from different hubs that have a wide array of local contexts (rural vs. urban), varying financial resources, and differing roles at their CTSA (ex. CTSA Evaluators vs. Administrators).

Concept mapping is one approach to addressing these challenges by enabling stakeholders to define evaluation measures collaboratively through an asynchronous participatory platform, thereby fostering a quantifiable consensus and shared vision while respecting individual hub and institutional contexts (6–9). By design, this methodology is an example of participatory evaluation. This study utilized concept mapping to identify a comprehensive set of specific measures for evaluating the CTSA Program's success in meeting its goals. Input was gathered from a diverse range of perspectives and locations, spanning multiple hubs nationwide and involving over 100 key stakeholders, including CTSA Administrators, CTSA Evaluators, and NCATS staff. By engaging CTSA participants from a set of different but complimentary roles, the study sought to uncover areas of consensus or disagreement around key themes while ensuring differing perspectives were represented.

## 2 Materials and methods

Concept mapping is a mixed-methods approach that applies quantitative analysis to qualitative inputs. This methodology was chosen for this project as opposed to other analogous approaches such as the Delphi Method (10, 11) or Nominal Group Technique (NGT) (10, 12) because: multiple non geo-located stakeholders needed to asynchronously and collaboratively define and organize ideas; both qualitative and quantitative analysis was preferable for structuring a variety of concepts; visualization of conceptual relationships would be more useful than simple ranking; and group consensus-building was a key goal for the process overall. As Trochim describes it, concept mapping is “…*a unique integration of qualitative (group process brainstorming unstructured sorting interpretation) and quantitative (multidimensional scaling hierarchical cluster analysis) methods designed to enable a group of people to articulate and depict graphically a coherent conceptual framework or model of any topic or issue of interest*” [(13), p. 166]. This method has been used extensively in planning and evaluation since the 1980s (6, 9, 13, 14), and involves four essential components detailed below: Participant Selection, Data Collection, Analysis, and Interpretation.

Implementing this methodology required three waves of primary data collection and participant engagement to interpret findings. All waves of data collection involved soliciting volunteers at regularly scheduled CTSA Administrator and Evaluators meetings, and internal meetings of NCATS staff as well as sending emails directly to these targeted audiences for participation. These three groups of stakeholders—CTSA Administrators, CTSA Evaluators, and NCATS staff—were non-randomly sampled for heterogeneity. More specifically, they were also selected because of their direct and often complementary roles in designing, implementing, and utilizing evaluation data to monitor and convey the value-add and impact of CTSA-funded activities. Participation was voluntary and each participant did not need to participate in all three waves of data collection.

The first wave of data collection involved the brainstorming of measures where participants were asked to respond to a focus prompt with a data collection instrument that was created in REDCap. After providing the CTSA program goals, the data collection instrument included the following focus prompt to guide participants: “Please brainstorm as many measures as you can in response to the following prompt: ‘*One specific measure I think should be used in an evaluation of the CTSA program is…*.” Data collection opened on February 8, 2022 and closed on March 8, 2022. A total of 320 statements were collected from participants. While the focus prompt specifically solicited measures, some participants gave statements about measures instead. Therefore, we refer to the raw data that was collected as “statements.” It is interesting to note that select non-NCATS/NIH staff were invited to participate (*N* = 3), but ultimately did not participate in any waves of data collection.

The next step involves unitizing the statements, a content analysis methodology (15) that is part of the concept mapping process. Trochim et al. (16) describe “unitizing” as “…the process of dividing a continuous text into smaller units that can then be analyzed” (P. 67). For example, there were instances where responses were double-barreled (e.g., “Describe the impact of CTSA funding on community health or translation into clinical practice”). These responses were then parsed out by two of the authors (CK and JD) into single idea statements (e.g., “Describe the impact of CTSA funding on community health” and “Describe the impact of CTSA funding on translation into clinical practice”). Of the 320 statements originally submitted by participants, statements were then parsed into 499 single idea statements. Two authors (CK and JD) then iteratively and inductively combined these single idea statements into 81 final statements that were used for the remainder of the process and constitute the detailed content of the mapping exercise. More formally, the authors use an inductive and independent blind coding process where similarities between statements arose from the data itself (induction) rather than having a pre-determined list of categories, bins, or statements for which each of the 499 single statements would need to be combined (deduction). The process was also “iterative” in that the 499 single idea statements were exchanged iteratively with two authors until the final list of statements was obtained. This approach, which combines iterative and inductive processes, can be described as inductive content analysis (17). A flow diagram of the brainstorming data collection and arrival of the final statement set for sorting is available in Supplementary Figure S1.

The second wave of data collection involved soliciting the same three groups—CTSA Evaluators, Administrators, and NCATS staff—to sort the 81 measures. For the sorting, participants were given a macro-enabled spreadsheet (18) and asked to assign labels of their choosing next to each statement. The only restrictions that were given to the participant were as follows: (1) spreadsheets could not be reformatted in any way, (2) each statement could only be labeled exclusively in one group, and (3) all statements could not be put into a single group. It is important to mention that while the approach used involves having participants “label” each statement, this is functionally the same as having them physically sort similar statements into piles that are then labeled (ex. “Collaborations”, “Translation Measure”, “Success Stories”, etc.). All submitted labels were later used in a subsequent wave of qualitative analysis to assign representative titles to the clusters in the concept map. Data collection for the sorting part of this activity opened on February 20, 2023, and closed on March 3, 2023. Three participants submitted their sorted statements after the due date (two NCATS staff and a participant from a CTSA hub), with the last submitted received on April 10, 2023. These late submissions were included in the analysis.

Shortly following the sorting activity, the groups were then asked to rate the 81 measures by their feasibility and importance using a REDCap form. More specifically, participants were asked to rate measures on a five-point scale according to their relative feasibility of collection and relative importance for assessing the extent to which the CTSA program is meeting its goals, where: 1 = Not Feasible/Relatively Unimportant; 2 = Somewhat Feasible/Important; 3 = Moderately Feasible/Important; 4 = Very Feasible/Important; and 5 = Extremely Feasible/Important. Participants were asked to spread out their ratings and try to use each of the five rating values at least several times.

The RCMap package in R was used to perform all analyses (19). The analysis begins with the construction from the pile-sorting information of an NxN binary symmetric matrix of similarities, *X*__*ij*_, k_, for each sorter. For a single participant (indexed by *k*) *X*__*ij*_, k_ = 1 for any two statements i and j, if the two items were placed together in the same pile (category label) by the participant, otherwise a 0 is entered. The total similarity matrix is obtained by summing across all individual participants' matrices (20). Therefore, each cell in this total matrix indicates how many participants sorted the two statements together (regardless of what other statements they may have been sorted with). This total similarity matrix is the input for nonmetric multidimensional scaling (MDS) with a two-dimensional solution, which yields a two-dimensional (x,y) configuration/plot of the statements such that statements that were piled together more frequently are located closer to each other in this space while statements piled together less frequently are further apart (the “point map”). This x,y configuration is the input for hierarchical cluster analysis using Ward's method (21) which effectively partitions the x,y configuration of statements into non-overlapping clusters, called the “cluster map”. The importance and feasibility rating data are averaged across persons for each statement and cluster in a second stage of analysis described below.

Once the basic map structure is determined it is possible to construct any number of pattern match graphs [also called “ladder graphs”, and known in the field of data visualization as a parallel coordinates graph (22)] that either compare two ratings (for all participants or any subgroups) or two groups (for any rating). Groups were determined from the demographic data that was collected. A pattern match or ladder graph is a useful visual device for showing relationships and especially for highlighting the degree of relationship between the entities being displayed. A Bonferroni correction was applied to the differences in means tests reported in the ladder graphs due to the multiple hypothesis tests performed. Finally, a “Go-Zone” plot (13) was generated to visually summarize feasibility and importance measures across all raters. Quadrants for each Go-Zone were generated using overall mean ratings for feasibility and importance, respectively.

The final step of the Concept Mapping process requires engagement with representative stakeholders, which we refer to as the Interpretation Group, to respond to the general layout of the concept map and associated visualizations. This group is tasked with providing final high-level feedback on the graphic representations based on the analysis described above, as well as a review of the concept map cluster titles based on qualitative coding of label names aggregated across all sorting participants Given the hierarchical nature of the relationship between funders and grant recipients, we prioritized capturing this final wave of targeted feedback strictly from the perspective of the hubs (Administrators and Evaluators). A stratified random sample of 12 participants was taken from the list of raters, with role (CTSA Administrator vs. Evaluator) and CTSA hub size (Small, Medium, and Large according to budgeted direct costs of the hub award) as strata (6 evaluators and 6 administrators, with two from each hub size within each sub-strata). Of the 12 participants, 4 did not respond or declined to participate in the interpretation step (2 evaluators and 2 administrators from medium and large hubs). Hubs that already had a participant committed to interpreting the findings were removed from the rating list for resampling, and additional potential participants were then selected. These participants had characteristics that were the same as those who did not respond or declined. All 4 of the newly sampled participants agreed to attend the interpretation session, and only one did not attend the actual meeting (an evaluator from a large hub). NCATS staff did not participate in the interpretation session.

## 3 Results

Table 1 shows the descriptive statistics of participant characteristics, as well as participating hubs. During the study period, there were 61 active CTSA hubs during the first wave of data collection (i.e., Brainstorming) and 65 active hubs during the second and third waves (i.e., Sorting and Rating). For the Brainstorming stage of Concept Mapping, we had participants from 20 out of 61 hubs (33%) and 33 participants, which included 8 NCATS staff. Participation increased with subsequent waves of data collection, with 44 out of 65 hubs participating in the Sorting stage (68%), and 47 out of 65 hubs (72%) participating in the Rating stage. The number of individual participants also increased with each stage, with a 124% increase (from *N* = 33 to *N* = 74) in the number of participants from Brainstorming to Sorting and a 36% increase (from *N* = 74 to *N* = 101) from Sorting to Rating. Across all waves of data collection, nearly half of all participating hubs were small in size, which roughly reflects the proportion of total hubs in the portfolio of that size (23). The largest group of participants in the sample across all phases of data collection were CTSA Evaluators.

**Table 1:** Descriptive statistics of participants.

**Hub characteristics**	**Phase 1: Brainstorming**		**Phase 2: Sorting**		**Phase 3: Rating**	
	**%**	***N***	**%**	***N***	**%**	***N***
**Size**						
Large	30%	6	32%	14	34%	16
Medium	15%	3	18%	8	15%	7
Small	55%	11	50%	22	51%	24
**Region**						
Midwest	30%	6	25%	11	21%	10
Northeast	20%	4	27%	12	34%	16
South	40%	8	34%	15	26%	12
West	10%	2	14%	6	19%	9
**Number of participants**						
1	75%	15	66%	29	57%	27
2	25%	5	20%	9	21%	10
3	0	0	7%	3	9%	4
4+	0	0	7%	3	13%	6
**Hub participant characteristics** **Size**						
Large	28%	7	34%	23	29%	26
Medium	20%	5	22%	15	19%	17
Small	52%	13	44%	30	53%	48
**Region**						
Midwest	36%	9	29%	20	18%	16
Northeast	20%	5	32%	22	30%	27
South	36%	9	25%	17	23%	21
West	8%	2	13%	9	30%	27
**Role**						
Evaluator	76%	19	71%	48	51%	46
Administrator	20%	5	15%	10	15%	14
KL2 PI	0%	0	0%	0	1%	1
TL1 PI	0%	0	0%	0	3%	3
UL1 PI	0%	0	0%	0	2%	2
Community partner	0%	0	0%	0	1%	1
Other CTSA hub staff	4%	1	9%	6	16%	15
Other	0	0	6%	4	10%	9
**Total hub participants only**		25		68		91
**NCATS staff**		8		6		10
**Total unique participants**		33		74		101
**Total unique hubs**		20		44		47

“Other CTSA Hub staff” for brainstorming was a staff member in Informatics. “Size” is defined by hub award direct cost. “Region” is defined by the US Census (https://www2.census.gov/geo/pdfs/maps-data/maps/reference/us_regdiv.pdf).

PI, Principal Investigator.

**Figure 1:**
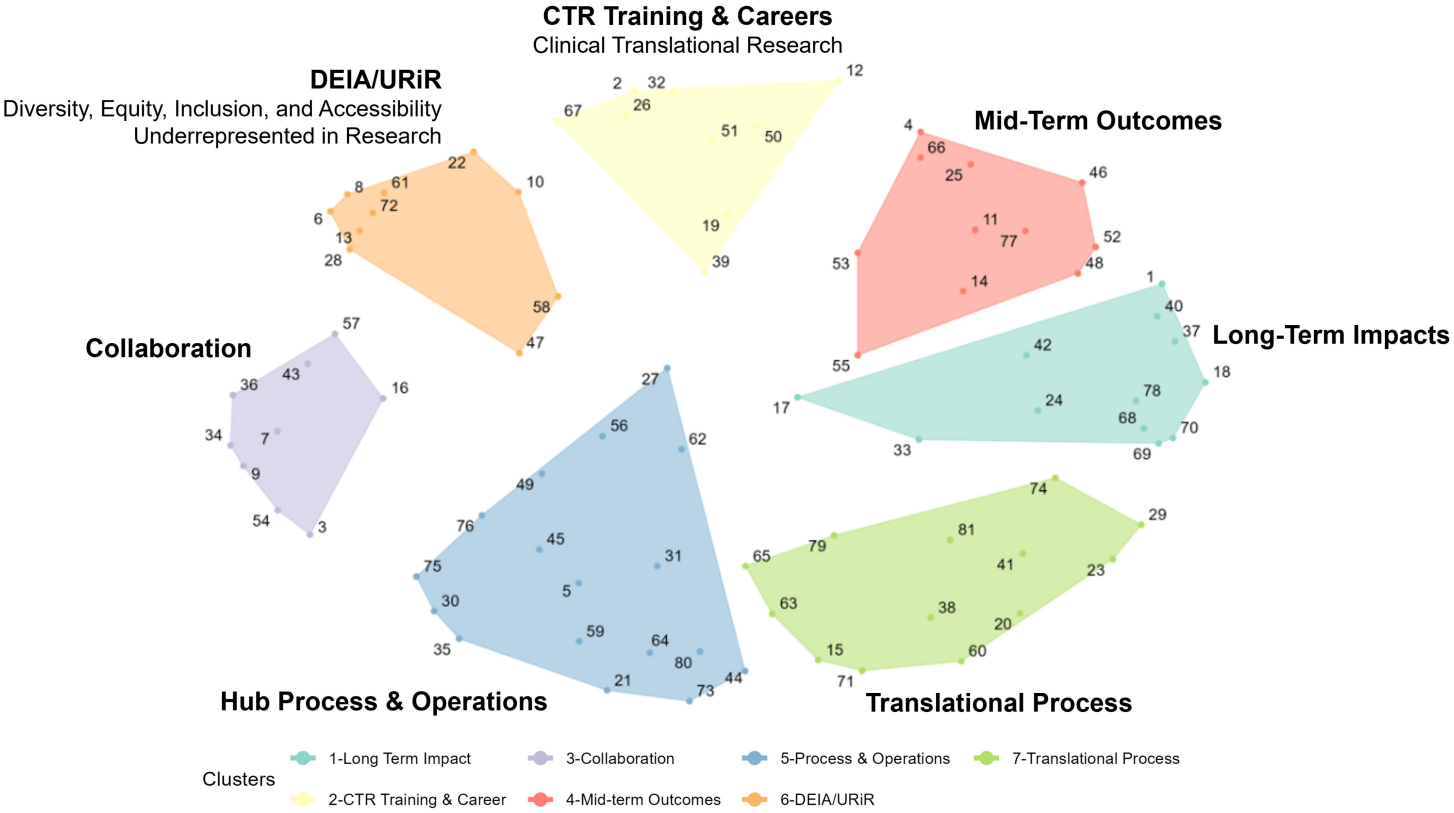
A concept map of CTSA measures.

Figure 1 shows the concept map (specifically, the “cluster map”), a graphic depiction of the composite thinking of all participants based on the cluster analysis, and Table 2 lists all 81 measures represented in the plot. The number of clusters (*K* = 8) was chosen by examining an “elbow plot” of the within sum of squares by the number of clusters (24). All eight statements in Cluster 8, located in the middle of the map as a “weak center”, were recoded to adjacent clusters based on manual review of the statements by the authors (CK and JD). Statements 27, 56, and 62 were recoded to Cluster 5 (Hub Processes and Operations); Statements 47 and 58 were recoded to Cluster 6 [*Diversity, Equity, Inclusion, and Accessibility (DEIA)/Underrepresented in Research (URiR)*]; Statements 17 and 55 were recoded to Cluster 4 (*Mid-Term Outcomes*), and Statement #33 to Cluster 1 (*Long-Term Impacts*).

**Table 2:** Concept mapping measures.

**Statement**	**Cluster**	**Statement**	**GoZone category**	**Mean importance**	**Mean feasibility**
1	1	Number and type of patents or trademarks filed and/or received (e.g., IP data, implementation science etc.)	High feasibility low importance	3	3.58
2	2	Number and type of trainings offered by Hub (e.g., courses, certificates, workshops, seminars, tracks, etc.)	High feasibility high importance	3.68	4.17
3	3	Institutional collaboration and commitment to clinical and translational science research (e.g., number of projects and protocols, in-kind support, $ and personnel)	Low feasibility high importance	3.59	3.07
4	4	Number of pilot grants advancing to clinical trial proposals and/or awards	High feasibility high importance	3.86	3.92
5	5	Median time to complete CTSA Hub-supported consultation and/or services (duration in days)	Low feasibility low importance	2.51	3.08
6	6	Number and type of measurable plans, policies or changes related to diversity, equity, and inclusion	Low feasibility high importance	3.74	2.68
7	3	Number and types of CTSA Hub interactions with state, local and public health entities	Low feasibility low importance	3	2.67
8	6	Qualitative data regarding how gender and racial diversity in clinical translational research can be achieved and/or what is needed	Low feasibility high importance	3.7	2.66
9	3	Number and types of new or ongoing collaborations with multiple CTSA Hubs and/or national consortium	High feasibility high importance	3.59	3.65
10	6	Number and percent of pilot awardees overall and by relevant demographics (e.g., women and underrepresented populations)	High feasibility high importance	3.64	4.23
11	4	For newly emerging health crises requiring a rapid response: Number of CTSA-affiliated investigators publishing relevant results within X period of time (in months or years)	High feasibility low importance	3.23	3.24
12	2	Relative familiarity with the term “translational science” among key indicator groups (healthcare providers, leaders of relevant community organizations and academic faculty in relevant fields)	Low feasibility low importance	2.69	2.36
13	6	Number and type of underrepresented populations in clinical trials tool	High feasibility high importance	4.14	3.34
14	4	Number and type of CTSA Hub supported services with subsequent grants and/or publications cited	High Feasibility High Importance	3.52	3.26
15	7	Number of different CTSA initiatives with a specific focus on >1 of the following: quality, safety, efficiency and effectiveness of clinical research	Low feasibility low importance	2.99	2.88
16	3	Number, type, duration, and quality of Hub-supported community engagement services and tools	High feasibility high importance	3.64	3.26
17	1	Number of datasets made discoverable as a result of Hub-supported Informatics resources	High feasibility high importance	3.28	3.29
18	1	The collection of high-level success stories (e.g., novel approaches or collaborations, mitigating translational science roadblocks)	High feasibility high importance	3.85	3.29
19	2	Number and type of changes in promotion and tenure policy as the result of Hub activities	Low feasibility low importance	2.87	2.37
20	7	Number of Hub supported opportunities created for novel care approaches for clinical research participants relative to other regional providers	Low feasibility low importance	2.87	1.96
21	5	Cost per participant enrolled in NIH-supported clinical trials	Low feasibility low importance	2.69	2.39
22	6	Scientific interdisciplinarity as measured by number and type of Doctoral/MBA/MPH degree types, scientific areas, and/or collaborations between and support of various departments, in Hub-supported work	Low feasibility low importance	2.86	2.77
23	7	Return on investment (ROI): The timing and magnitude of expected total gains relative to the timing and magnitude of expected total costs	Low feasibility low importance	3.2	2.24
24	1	Systematic reviews and/or meta evaluation (e.g., the Cochrane or Campbell Collaboration showing the reproducibility of results published in a discipline over a set time period)	Low feasibility low importance	2.75	2.64
25	4	Number and/or percent of Hub-supported Pilot projects with >1 subsequent publication	High feasibility low importance	3.16	4.15
26	2	Number of and satisfaction with formal mentors supported by the Hub	High feasibility high importance	3.3	3.53
27	5	Types of research projects supported by Hub (e.g., T1–T4, various disciplines)	High feasibility high importance	3.3	3.64
28	6	Proportion of CTSA-affiliated investigators who request or receive CTSA Hub-supported materials in a language other than English	Low feasibility low importance	2.42	2.73
29	7	The use of retrospective case studies	Low feasibility low importance	2.57	2.88
30	5	Number and type of active multi-center trials	High feasibility high importance	3.39	3.95
31	5	Qualitative measures of Hub services quality (satisfaction, reliability, responsiveness)	High feasibility high importance	3.43	3.27
32	2	Number and types of knowledge and/or skills attained by participants of trainings offered by Hubs	Low feasibility high importance	3.44	3.02
33	1	Number and type of new programs as a result of CTSA Hub activity (e.g., entrepreneurship, translational science.)	High feasibility high importance	3.4	3.31
34	3	Number and types of collaborative research projects and collaborators within a CTSA Hub	High feasibility high importance	3.52	3.19
35	5	Number and type of available infrastructure or resources for multi-site clinical trials per CTSA Hub (e.g., Access to underrepresented populations, Clinical Trials Management Systems/EHR, etc.)	High feasibility high importance	3.46	3.44
36	3	Number and types of Hub collaborations with community members, advisors or partner agencies	High feasibility high importance	3.85	3.37
37	1	CTSA Hub-level listing of scientific and operational innovations developed, demonstrated, and disseminated	Low feasibility high importance	3.82	2.88
38	7	Number and type of actions generated from RPPR review within a CTSA Hub	Low feasibility low importance	2.44	2.95
39	2	Number of CTSA hub website page views	High feasibility low importance	2.04	4.51
40	1	Number and type of award-winning innovations developed at CTSA Hubs	High feasibility low importance	3.13	3.24
41	7	Tracking Hub-supported research from one step to the next on the translational spectrum (T1, T2, T3, etc.) via operational markers (e.g., first in human, clinical trial phases, FDA approval, etc.)	Low feasibility high importance	3.47	2.28
42	1	Number of FDA approvals received by CTSA Hubs	High feasibility low importance	3.16	3.78
43	3	Qualitative data regarding community experiences with and perceptions of research (trust, community value, equity, researcher preparedness, and indicators of successful engagement) and perceptions of optimism regarding positive health outcomes	Low feasibility high importance	3.89	2.83
44	5	Duration (raw number or median) in days from start to finish of IRB application (Common Metric: Could also be applied for sIRB or eSRC)	High feasibility low importance	3.18	3.95
45	5	Number and type of CTSA Hub supported service consultations and services	High feasibility low importance	3.19	4.05
46	4	Frequency and reach of CTSA-affiliated personnel interviewed by the media	Low feasibility low importance	2.18	2.64
47	6	Number of researchers served by the CTSA Hub (overall and by percent of relevant demographics e.g., women and Underrepresented Populations)	High feasibility high importance	3.53	3.41
48	4	The Altmetric Attention Score (a weighted count of all of the online attention discoverable for an individual research output, including but not limited to social media, news, and policy documents)	High feasibility low importance	2.7	3.19
49	5	Tracking number of new Hub personnel (e.g., tracking key personnel changes over time, turnover rate)	High feasibility low importance	2.51	3.63
50	2	Number, type and percent of career impacts on participants in Hub-supported career development (e.g., promotion, subsequent funding, leadership)	Low feasibility high importance	3.69	3.02
51	2	Number and percent of trainees and scholars who remain engaged in research after training (Common Metric: Overall and by relevant demographics e.g. women, underrepresented populations, etc.)	High feasibility high importance	3.87	3.51
52	4	Bibliometrics, general (e.g., the wide range of bibliometrics used in academia such as the H-index or the Journal impact factor)	High feasibility low importance	2.97	3.75
53	4	Time from end of pilot grant to first subsequent grant and/or publication (duration in days)	High feasibility low importance	2.85	3.14
54	3	Team Science readiness regarding issues such as Authorship & Credit; Contingencies & Communicating; and Conflict of Interest (e.g., Checklist published by the National Cancer Institute)	Low feasibility low importance	2.98	2.41
55	4	Number and type of NIH institutes or programs (outside of NCATS) using CTSA resources or CTSA developed resources	Low feasibility low importance	2.99	2.39
56	5	Number and type of Hub-supported faculty involved in clinical research	High feasibility low importance	3.19	3.57
57	3	Number and type of community members trained by Hubs	High feasibility high importance	3.45	3.28
58	6	Number and types of CTSA Hub supported research studies (e.g., those involving health disparities or special populations)	High feasibility high importance	3.72	3.61
59	5	Number (and growth in number) of patients enrolled into Hub supported trials	High feasibility high importance	3.48	3.58
60	7	Measures of data quality including performance data readability, relevance, reliability, representative, and reproducibility in Hub supported research	Low feasibility high importance	3.39	2.3
61	6	Proportion of positions representing individuals from underrepresented populations in research across the biomedical workforce (i.e., coordinators, technicians, analysts, not just investigators, or Hub leadership)	Low feasibility high importance	3.57	2.58
62	5	Number of new and repeat investigators receiving CTSA Hub services	High feasibility low importance	3.06	3.9
63	7	Reduced number of deferrals in CTSA Hub-supported research projects	Low feasibility low importance	2.33	2.51
64	5	Time to activation of new clinical trials supported by the CTSA Hub (in days)	High feasibility high importance	3.26	3.42
65	7	Qualitative data regarding number and kinds of barriers faced by Hubs	Low feasibility high importance	3.57	2.87
66	4	Number and/or percent of Hub supported Pilot projects with >1 subsequent grant for extramural funding	High feasibility high importance	3.32	3.73
67	2	Number and percent of KL2 and/or TL1 applicants, participants and graduates (overall and by relevant demographics, e.g., women, underrepresented populations, etc.)	High feasibility high importance	3.93	4.4
68	1	Number and type of Clinical and Medical Benefits (from Translational Science Benefits Model. e.g. procedures, guidelines, tools, and products)	Low feasibility high importance	3.71	2.66
69	1	Number and type of Economic Benefits (from Translational Science Benefits Model. e.g., commercial products, financial savings and benefits)	Low feasibility high importance	3.43	2.35
70	1	Number and type of Community and Public Health Benefits (from Translational Science Benefits Model. e.g., health activities, care, and promotion)	Low feasibility high importance	3.68	2.51
71	7	Changes made at a Hub in response to Rapid Cycle Quality Improvement (RCQI) by theme and percentage change	Low feasibility low importance	2.69	2.25
72	6	Quantitative measures of CTSA leadership, staff, and supporting institution and/or catchment area by demographic diversity (Underrepresented populations, gender, early-career, socioeconomic status, etc.)	Low feasibility high importance	3.36	3.04
73	5	Clinical trial process quality (e.g., number of audits, monitoring or biorepositories, adherence to FDA requirements)	Low feasibility high importance	3.25	2.96
74	7	Number of outcomes and innovations of CTSA supported and/or funded clinical research (e.g., quality, safety, efficacy, clinical and behavioral innovations and/or outcomes, IND, etc.)	Low feasibility high importance	3.67	2.63
75	5	Number of CTSA Hubs integrating EHR data (i.e., feasibility, recruitment)	High feasibility high importance	3.37	3.52
76	5	Average time to fill clinical research professionals (CRP) positions relative to other, readily available staff positions (duration in days)	Low feasibility low importance	2.64	2.64
77	4	Number of publications by CTSA Hub-affiliated authors overall, and percent by authors demographics (e.g. gender and underrepresented populations, field...)	High feasibility low importance	3.17	3.43
78	1	Number and type of Policy and Legislative Benefits (from Translational Science Benefits Model. E.g., advisory activities, policies and legislation)	Low feasibility high importance	3.4	2.51
79	7	Proportion of phase 1 clinical trials (T1) to bench studies (T0) supported by the CTSA Hub	Low feasibility low importance	2.79	3
80	5	Number or percent of studies reaching the median accrual ratio (Common Metric: percent of participants accrued divided by percent of recruitment period to date)	Low feasibility low importance	3.16	2.97
81	7	Number of Hubs reporting on activities within a measurable period of time once an urgent public health need is identified for the national CTSA consortium (readiness/rapid response in days or months within specified categories, e.g., research, education, training, community engagement etc.)	Low feasibility high importance	3.47	2.8

Given the content of the focus prompt, this map suggests a structured, comprehensive framework to discuss and assess various potential measures to be used to evaluate whether the CTSA program is meeting its stated goals. Reading from left to right and moving clockwise on the map, the clusters can be described as follows:

- **Hub Process and Operations:** This cluster focuses on the operational aspects of CTSA hubs, including measures like time (in days) to complete consultations, the number and types of multi-center trials supported, and the quality of services.
- **Collaboration:** This cluster examines the collaborative efforts within and between CTSA hubs, including partnerships with community members, state and local public health entities, and other CTSA hubs. It also includes measures such as tracking qualitative data on community perceptions and experiences with research.
- **Diversity, Equity, Inclusion, and Accessibility (DEIA)/Underrepresented in Research (URiR):** This cluster includes measures around the integration of diversity and inclusion in clinical research, with examples such as the relative availability of research materials in different languages and the participation of underrepresented populations in clinical trials.
- **Clinical Translational Research (CTR) Training & Careers:** These measures focus on training and career development. This cluster includes measures such as the number of training programs, overall mentorship satisfaction, specific career outcomes, and the retention of trainees in research.
- **Mid-Term Outcomes:** This cluster aims to track mid-term progress such as the more proximal impact of hub-supported projects on subsequent publications or grants, bibliometric indicators or changes in promotion and tenure policies.
- **Long-Term Impacts:** This cluster includes measures for the broad, long-term effects of CTSA activities, such as the number of Food and Drug Administration (FDA) approvals, economic and community health benefits, and/or policy or legislative impacts. It also contains measures around systematic reviews and high-level success stories. Notably, all Translational Science Benefits Model (TSBM) measures (numbers 68, 69, 70, and 78) were sorted thematically into this cluster.
- **Translational Process:** This cluster concentrates on tracking the progression of research from the early stages of discovery to clinical application. Measures include the use of retrospective case studies, or the tracking of research across the translational spectrum (e.g., from bench to clinical trials).

**Figure 2:**
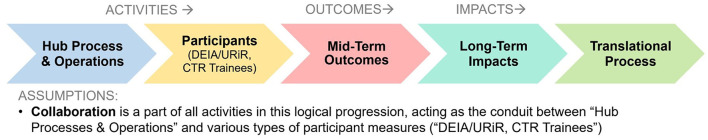
CTSA evaluation concept map clusters arranged as a logical process model.

Figure 2 shows a logical progression with the clusters from our map in Figure 1 “flattened” and listed in temporal order. Working clockwise from the bottom of the map in Figure 1, we begin the process in Figure 2 by first listing CTSA Activities such as *Hub Processes and Operations (5)* (how hubs carry out their work). We can compress the next two clusters in the map: *Diversity, Equity, Inclusion, and Accessibility (DEIA)/Underrepresented in Research (URiR; 6) and CTR Training & Careers (2)* into a single common phase in Figure 2 focused on key Participants, with *Collaboration (3)* as a critical component for all relevant participants in the Activities phases. Continuing clockwise around Figure 1 to inform the process phases in Figure 2, we can next list clusters for Outcomes *Mid-term Outcomes (4)* (immediately observable intermediate results) followed by *Long-term Impacts (1)* (final consequences or effects) and finally *Translational Processes (7)* (the ultimate mission of the CTSAs, to move research from discovery to application). Thus, whether viewed clockwise on the concept map (Figure 1) or as a simplified linear progression (Figure 2), our project participants logically grouped the measures related to CTSA Activities (formative evaluation) in specific logical relation to those corresponding to Outcomes and Impacts (summative evaluation). In reviewing both graphic depictions, we can see that project participants showed agreement not only in a comprehensive list of measures in general, but in a rational thematic framework for where these measures belonged relative to the evaluation of the CTSA program.

**Figure 3:**
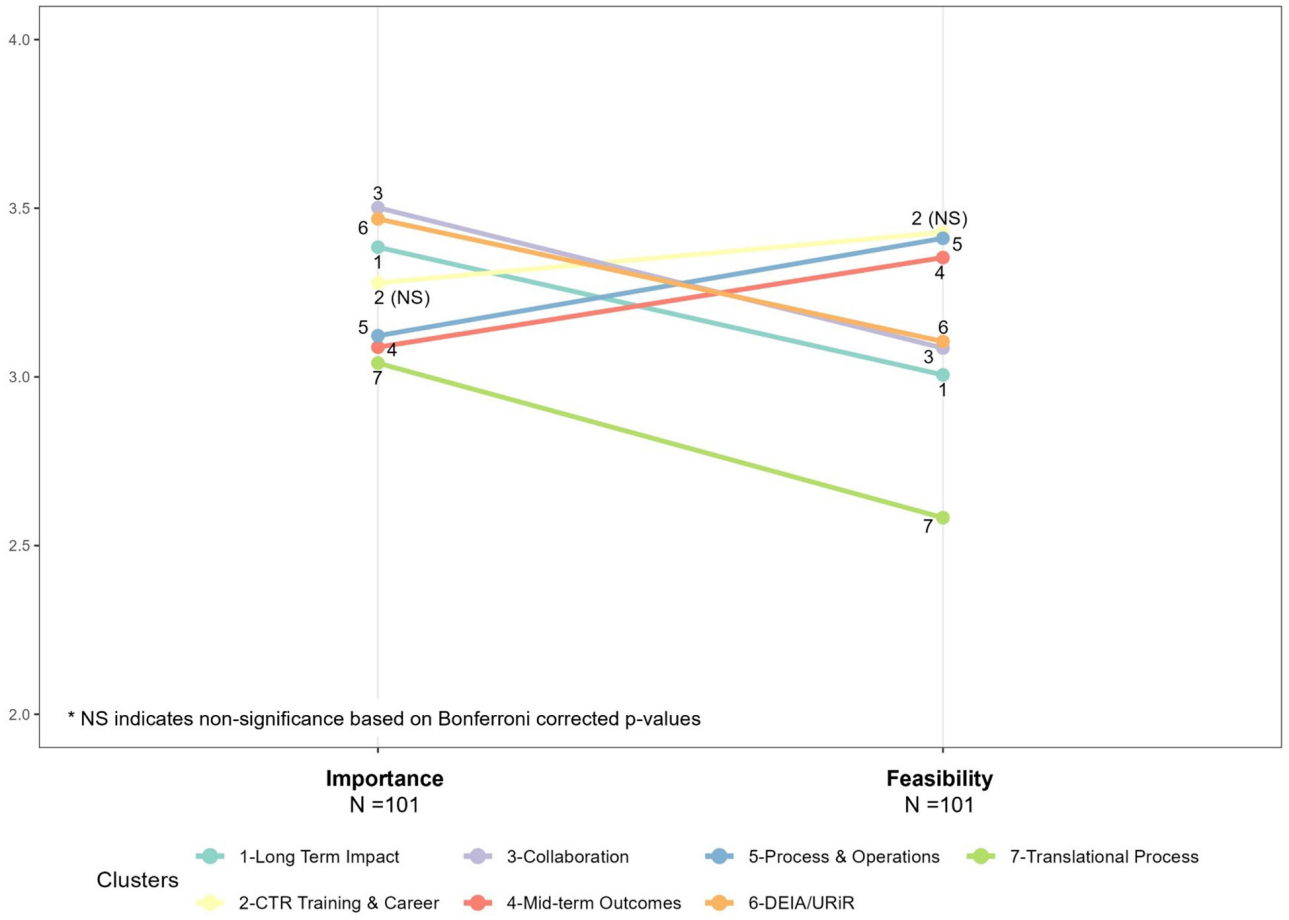
Parallel coordinates plots (or “ladder graphs”), mean feasibility and mean importance.

Figure 3 shows the parallel coordinates plots, or “ladder graphs”, which describe the relationship between feasibility and importance for the 81 measures. Bonferroni corrected *p*-values indicate that the mean feasibility and mean importance for all seven clusters were statistically different except for CTR Training and Careers (*p* = 0.009); but the magnitude of the difference varied by cluster. On average, more than half of the seven clusters of measures were rated as appreciably more important than feasible, with lower averages overall on the feasibility side of the figure. *Translational Process (7) and Long-Term Impacts measures (1)* stood out as the least feasible relative to their importance. Notable exceptions were *Mid-Term Outcomes (4)* and *Hub Processes and Operations (5)*, which were the only clusters rated as less important than feasible on average. Interestingly, these clusters are more closely tied to the daily activities of CTSA hubs and are frequently utilized for external reporting as well as for internal review by hub leadership.

**Figure 4:**
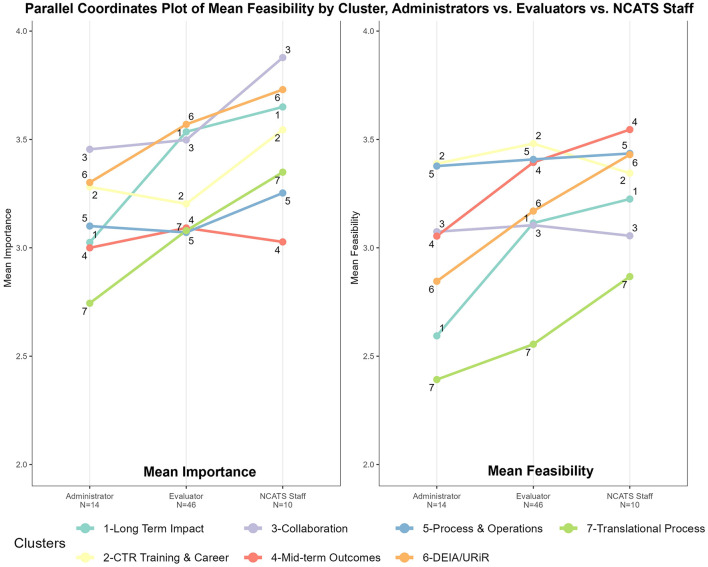
Feasibility vs. importance “ladder graphs” by primary participant type: CTSA administrators, CTSA evaluators, and NCATS staff.

Subgroup heterogeneity by participant type is evident when the data were disaggregated, as seen in Figure 4. Figure 4 (“ladder graphs”) stratifies average ratings for Mean Importance (left) and Mean Feasibility (right) by the three primary participant roles: CTSA Administrators, CTSA Evaluators, and NCATS staff. Here the pattern of high importance and low feasibility regarding *Long-Term Impacts (1)* can be seen in more relief when broken out by participant type. On the left of Figure 4, in regards to relative average importance, the steep angle of *Long-Term Impacts (1*) illustrates Administrators ranking this cluster in the bottom third, whereas Evaluators and NCATS staff ranked the same cluster in the top third. *Mid-term Outcomes (4)* and *Long-Term Impact (1)* represent the most pronounced, statistically different discrepancies in average importance ratings between CTSA Evaluators and Administrators. Moreover, both NCATS staff and CTSA Administrators had average importance ratings that were not statistically different from CTSA Evaluators on these two clusters. In contrast, NCATS staff rated almost all measures as more important on average than their peers in Administration and Evaluation in terms of the magnitude of their means, but were only statistically different from CTSA Evaluators on *Collaboration (3)*. On the right of Figure 4, regarding relative average feasibility, there are more steep lines illustrating differences observed by cluster between CTSA Administrators and NCATS staff, and a pattern of more agreement between the feasibility ratings of CTSA Evaluators and NCATS staff. The mean feasibility ratings between CTSA Evaluators and NCATS staff are not statistically different across nearly all clusters of measures except for *Translational Process (7)* shown at the bottom of the graph. Overall, these trends in Figure 4, along with low Bonferroni-corrected *p*-values—show the most measurable disagreement in the feasibility ratings for CTSA Administrators vs. the other two subgroups (CTSA Evaluators and NCATS staff). The Significance Tests for Ladder Graphs by Group are shown in Table 3.

**Table 3:** Significance tests for ladder graphs by group.

	**Importance**			**Feasibility**		
**Group**	**Evaluators vs. NCATS**	**Evaluators vs. administrators**	**Administrators vs. NCATS**	**Evaluators vs. NCATS**	**Evaluators vs. administrators**	**Administrators vs. NCATS**
1	0.24	*p* < 0.0001^*^	*p* < 0.0001^*^	0.24	*p* < 0.0001^*^	*p* < 0.0001^*^
2	0.01	0.54	0.12	0.28	0.49	0.79
3	0.0001^*^	0.70	0.00	0.65	0.81	0.90
4	0.54	0.39	0.84	0.17	0.004^*^	0.001^*^
5	0.04	0.73	0.16	0.75	0.74	0.61
6	0.13	0.02	0.002^*^	0.02	0.01	0.0001^*^
7	0.02	0.001^*^	*p* < 0.0001	0.001^*^	0.09	*p* < 0.0001^*^

* Significance thresholds are based on Bonferroni-corrected p-value threshold of 0.007.

**Figure 5:**
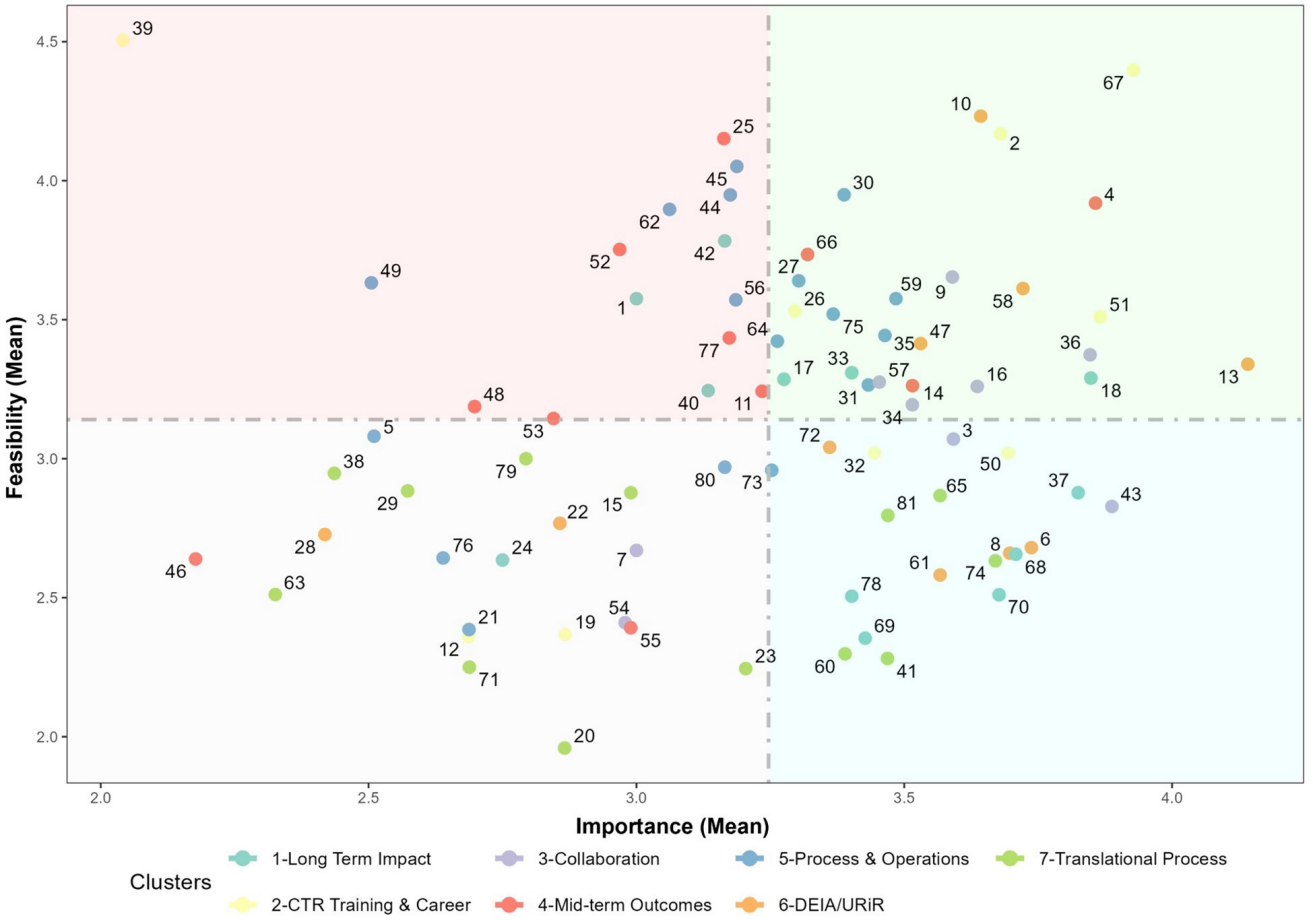
Feasibility vs. importance “go-zone” plot for all concept mapping measures.

Figure 5 visually represents all measures as single points based on their average Feasibility and Importance (“GoZone plot”). Measures with highly rated importance and feasibility can be seen as points in the upper right side of the Figure, whereas measures with relatively low importance and feasibility can be seen as points on the lower left. High Feasibility-Low Importance and Low Feasibility-High Importance measures appear in the upper left and lower right, respectively. The lower right quadrant—Low Feasibility-High Importance measures—is of particular interest with respect to the differing rating levels between Administrators, Evaluators and NCATS staff especially in regard to *Long-Term Impacts (1)* and TSBM. For instance, measures 68 (Number and type of Clinical and Medical Benefits), 69 (Number and type of Economic Benefits), 70 (Number and type of Community and Public Health Benefits), and 78 (Number and type of Policy and Legislative Benefits) directly reference the full scope of the TSBM, a framework gaining popularity across the CTSA consortium (25, 26). *Translational Processes (7)* is also well-represented in the lower-right quadrant, with measures such as data quality (Statement 60), CTSA hub-level listing of scientific and operational innovations developed, demonstrated and disseminated (Statement 37), tracking hub-supported research from one step to the next on the translational spectrum (Statement 41), the number of outcomes and innovations of CTSA supported and/or funded clinical research (Statement 74) rated, on average, as having high importance but low feasibility.

## 4 Discussion

The purpose of this study was to conduct a comprehensive analysis of the range of measures for assessing the CTSA program's goals and to identify areas of consensus and differing perspectives. This effort resulted in three main findings. First, the concept mapping activity yielded a broad range of measures (*N* = 81). In terms of the overall volume of statements per themed cluster, *Process and Operations* had the greatest number of measures (>50% larger than the median). However, it stands to reason that in any large and complex program such as the CTSA, it is likely that causality will operate through many multiple pathways (the intentional use of multiple processes) toward a common goal (a smaller focused set of desired outcomes and impacts). Second, the clusters in our concept map corresponded with the components of a traditional logic model, illustrating the expected progression from actions to outcomes. Measures focused on CTSA activities and processes are included in the clusters represented on the left side of the map, and progress to measures associated with outcomes and impacts on right side of the map. A related finding was the TSBM measures were arrayed in a tight configuration on the far-right side of the map. This spatial placement and consolidation suggest that many participants classified the TSBM measures similarly. Third, the analyses stratified by role (in Figure 4: “ladder graphs”) showed diverging views on importance and feasibility by participant role (CTSA Evaluator vs. CTSA Administrator vs. NCATS staff), particularly regarding *Long-term Impact* measures, which included the TSBM (four out of nine measures in the cluster). There was also a striking and widespread consensus on the overall *importance* of the long-term impact measures. Evaluators and NCATS staff in particular showed marked consensus on the importance of long-term measures, However, this agreement sharply contrasted with the pronounced disagreement regarding the *feasibility* of implementing these measures in practice, revealing a substantial divide among key stakeholders. Interestingly, the vast majority of long-term impact measures ranked with both highly importance and low feasibility centered almost exclusively on the TSBM (in Figure 5: GoZone plot).

The patterns in these findings could be due to several factors. The discrepancies in perceived feasibility and the heavy representation of processes and operations measures could reflect functional differences in day-to-day responsibilities and scopes of work across roles. For example, *Hub Processes and Operations* was the cluster with the largest number of measures. Many of these measures are linked to narratives reported in annual progress reports (Measure #45: “Number and type of CTSA hub supported service consultations and services”) and continuous quality improvement activities routinely conducted at most hubs [Measure #64: Time to activation of new clinical trials supported by the CTSA hub (in days)]. These measures lie at the intersection of work that CTSA Evaluators and Administrators perform each year. However, Administrators, specifically, must prioritize proximal process measures aligned with hub operations, whereas Evaluators often find themselves balancing short-term programmatic reporting and deliverables with broader, hypothesis-driven questions NCATS staff are required to monitor program performance and across the consortium. Another consideration is that the contrasting views on feasibility by roles may reflect overall familiarity with measures and their implementation. For instance, in the concept mapping interpretation group, one CTSA Administrator expressed concern that measuring “*Number and Type of Economic or Public Health Benefits*” in their catchment area would be challenging. They wondered how to access economic data and grappled with the complexities of attribution vs. contribution. Meanwhile, in the same meeting, an Evaluator shared how their team already used several TSBM survey questions, collected through trainee applications, exit interviews and alumni surveys to gather high-level self-reported data. This highlighted a contrast: one side believed they needed sensitive, detailed financial measures in order to operate within the TSBM framework, while the other had already integrated straightforward self-reported surveys to capture essential data. In this short meeting, the Administrators learned that *both* types of data could be used within TSBM; broad economic indicators and individual success stories both fit in this flexible framework for measuring impact.

It should be noted that this concept mapping study had several limitations that must be considered when interpreting its findings. First, the concept mapping process relied on voluntary participation across the CTSA consortium, which may have introduced self-selection bias, as participants with stronger opinions or familiarity with evaluation practices may have been more likely to contribute. Second, the majority of participants were drawing on the perspective of a single hub (Evaluators and Administrators), and the total number of NCATS staff was relatively low. This means that the greater part of the feedback stemmed from a hub-specific rather than a consortium level perceptions and experiences. Third, all participants were part of the CTSA program in some manner, which may have introduced additional bias based on the preponderance of internal perspective. Fourth, while the RCMap package provided robust analytical tools for clustering and visualizing participant input, the manual reassignment of certain measures to specific clusters introduces a degree of subjectivity, potentially influencing the final cluster configurations. Fifth, given the rapidly evolving nature of translational science and the specific goals outlined in the NCATS Strategic Plan (27), the measures identified here may require regular updates to remain aligned with emerging priorities, technological advancements, and evolving program goals. Finally, in the specific context for this discussion, it is also important to note that concept mapping is a tool for illustrating the composite thinking of a diverse group at a single point in time, rather than a means for providing incontrovertible or static answers. For example, the feasibility and importance ratings illustrated in the Go-Zone charts and ladder graphs are based on subjective assessments at a single point in time, which may be influenced by respondents' individual experiences, familiarity with particular measures, and role-specific priorities.

Nevertheless, these findings suggest that there is no shortage of available measures to assess the CTSA programmatic goals, but there may be a lack of consensus on how to implement them effectively and efficiently. This opens opportunities for future work. These concept mapping results could support multiple complimentary frameworks such as a consortium-wide logic model and the TSBM. While individual CTSAs may have developed logic models to address local needs and individual grant aims (28), a logic model for the consortium has not yet been developed. Using the results of this concept map as a foundation for this model would have the benefit of being a “bottom up” and data-driven exercise representing the thinking of the wide range of participants as opposed to a “top down” exercise with authorship and buy-in limited to a minority of stakeholders. Simultaneously, these same findings highlight that while TSBM measures are currently recognized as highly important, there are significant challenges around perceptions on feasibility. This provides a focused starting point for developing strategies to address and overcome these barriers to evaluation implementation.

This project also revealed practical opportunities for NCATS to provide strategic leadership by integrating the interdisciplinary insights of Evaluators and Administrators. The concept mapping process and results from this analysis create a starting point for future collaborative evaluation activities centered on assessing the CTSA program and its progress toward achieving its goals. Just as there are many roads to Rome, there are many ways to support translation in clinical research. As reflected in the concept map, on the activities side of the logical progression we have numerous interventions and collaborations to support clinical translational research. By the time we get to the outcomes side of the logical progression we are essentially listing impact measures that revisit the central mission of the CTSA program: To increase the pace of development and availability of treatments; to enable more individuals and communities to contribute to and benefit from translational science; and to identify and address inefficiencies in translation that slow and even stop research efforts (27). To fully leverage the strengths of CTSA Evaluators, Administrators, and NCATS staff, it is essential to embrace their distinct roles and responsibilities. Administrators focus on monitoring their own hub's operations, NCATS oversee consortium-wide outcomes and impacts, and Evaluators bridge these perspectives, balancing program-level reporting with broader questions of long-term effects. These differences are not limitations, but integral features of the system's structure, presenting opportunities for collaboration to enhance the full breadth of evaluation of the CTSA program.

Ultimately, the most difficult and pressing work will not lie in the selection of measures, but in driving coordinated CTSA evaluation across the consortium. Frameworks like concept mapping, logic modeling, and TSBM offer concrete signposts on the “many roads to Rome”; but their utility in this navigation depends on coordinated direction. Of all three roles represented in this study, only NCATS has the unique perspective and operational authority to endorse a unified CTSA logic model associated with a specific set of impact measures. They are also the only contributors with the level of access and critical resources necessary to collect and analyze aggregated data for a program of this complexity, scale and importance. By using data from these findings to guide their ongoing efforts, NCATS can strengthen its ability to assess whether the CTSA is meeting its goals and demonstrate the program's broader value. If we are to overcome the roadblocks on the path to evaluation, there is an opportunity ahead to harness and align the unique perspectives and strengths of CTSA Evaluators, Administrators and NCATS consortium leadership. By setting a course centered around a shared vision for the way forward, these frameworks can guide us in the effective evaluation of the long-term impact of the CTSA Program.

## Data Availability

The raw data supporting the conclusions of this article will be made available by the authors, without undue reservation.
