# Supplementary figures and images for: Navigating the road ahead: using concept mapping to assess Clinical and Translational Science Award (CTSA) program goals
Source: Front Public Health. 2025 Mar 31;13:1562191. doi: 10.3389/fpubh.2025.1562191 (PMC11994586; doi:10.3389/fpubh.2025.1562191)

**Supplementary Figure 1:** Flow Diagram for Brainstorming Sample and Statement Set for Sorting

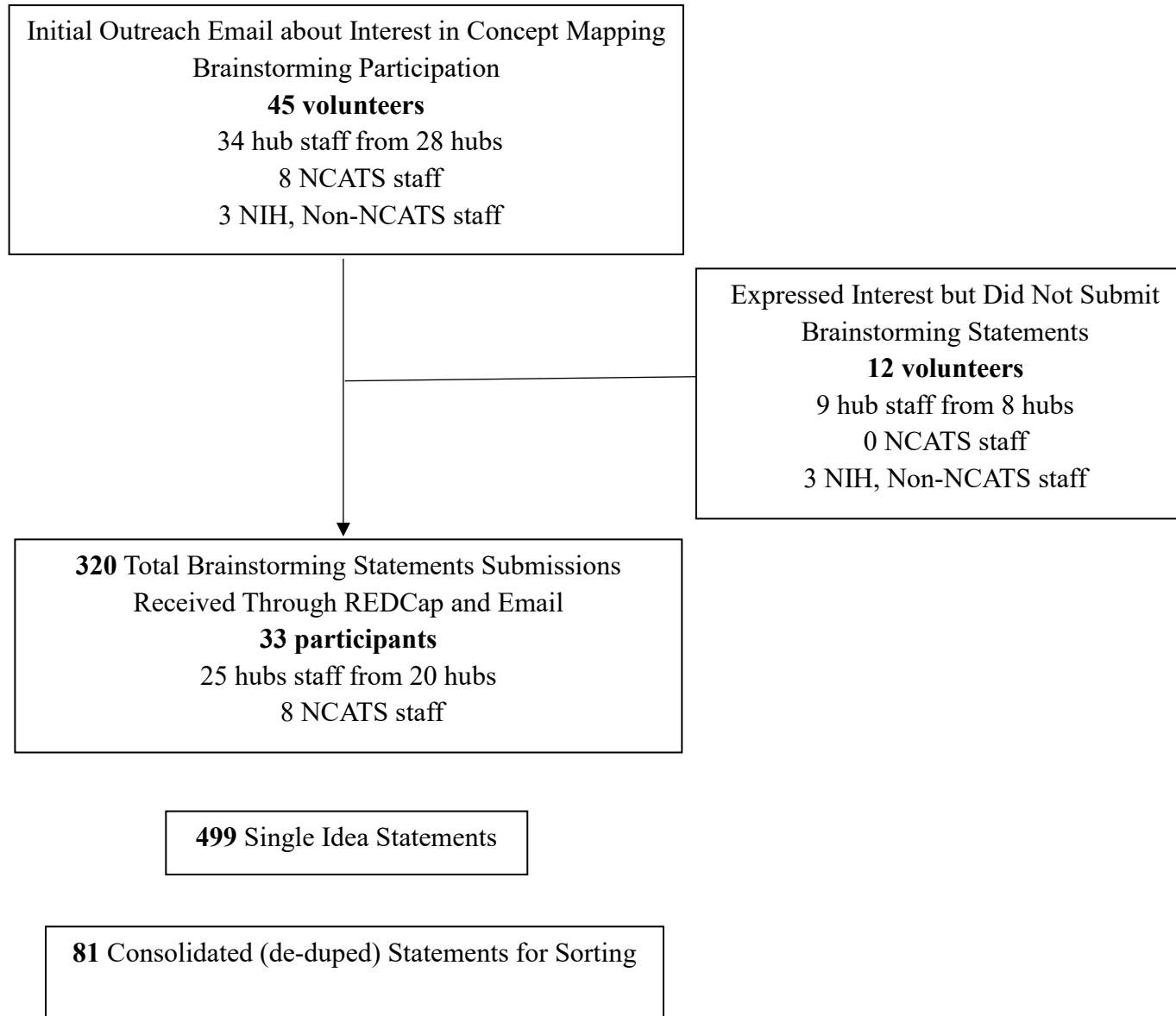

Supplement: Supplementary file 1 [file Image_1.pdf]
